# Zeppelin Raids: Fire Precautions at the London Hospital

**Published:** 1915-09-11

**Authors:** 


### ZEPPELIN RAIDS.

### Fire Precautions at the London Hospital.

At the quarterly court of the London Hospital held on Wednesday, September 1, the number of soldiers treated since the outbreak of the war was stated to be 2,984, of which 472 were Belgians. The total number of deaths among military patients amounted to forty. The work of treating the civilian population continues uninterruptedly.

The difficulty which has been experienced in retaining sufficient house physicians, house surgeons, and clinical assistants was explained, together with the arrangements made with the Admiralty and the War Office to overcome this difficulty, which have already been published in The Hospital. These arrangements have worked well, the young doctors have had the satisfaction of obtaining commissions, the hospital has retained their services, at any rate for three months, and the Navy and Army ultimately obtain medical men of more practical experience than would otherwise be the case.

Before making these arrangements the shortage of doctors became acute, and the War Office came to the assistance of the London Hospital authorities by seconding to the hospital seven Canadian doctors who came over with the Canadian contingent, and who volunteered for hospital work. These doctors remained at the hospital for a period of six weeks, and earned the hearty thanks of the Governors for the manner in which they readily accommodated themselves to the routine of the hospital, and for the highly efficient way in which they performed their duties.

"London" Men and Nurses in the War.

No less than 456 past and present students of the London Hospital are serving with the Navy and the Army. Of these, fifteen have been killed. The following honours have been obtained:

C.M.G.: Major Sir E. S. Worthington, M.V.O. D.S.O.: Lieut.-Colonel A. B. Soltau, Major J. G. Butler, Major A. C. Fox. Military Cross: Captain H. F. Vellacott, Lieut. E. J. Wyler, Lieut. R. A. Preston, Lieut. H. G. Winter. Mentioned in Despatches: Colonel Sir Bertrand Dawson, K.C.V.O., Colonel W. T. Lister, Major Sir E. S. Worthington, C.M.G., M.V.O., Captain E. C. Sprawson, Captain H. F. Vellacott, Captain H. G. Winter, Lieut. E. J. Wyler, Lieut. R. V. Dolbey, Lieut. R. Burgess, Lieut. A. J. Gilchrist, Lieut. A. B. Lindsay, Lieut. R. A. Preston, Lieut. L. C. Somerville, Lieut. H. F. Wolfenden.

One hundred and nineteen of the nursing staff of the London have joined the Services. The Royal Red Cross Decoration has been conferred on Miss Wainwright, Miss Elston, and Miss Richards, whilst Miss McCarthy, principal matron, has been twice mentioned in despatches. We are naturally proud of the fact that not only are the principal matrons in France and in the East both old "Londoners," but that Miss Becher, Matron-in-Chief of the Army Nursing Service, is also an old "Londoner."

Precautions Against Air Raids.

Special attention has been given by the committee to the precautions in hospital against fires which might be caused by Zeppelin raids. The porter staff is efficiently instructed in fire-drill, and an independent expert makes a quarterly inspection of all fire-appliances, and also witnesses drill and advises on the subject. On account of the exceptional risks special drills have been carried out, all hoses and apparatus have been subjected to severe practical tests, extra lengths of hose have been provided, and numerous extincteurs have been put in the wards, nurses' homes, etc.

To Centralise Financial Control.

As regards the internal management of the hospital the committee have decided to create a cashier's office, in which the payment and receipt of all moneys will be centralised, the rapid extension of the hospital in recent years having rather tended to decentralise the control. A sub-committee, consisting of Mr. W. T. Paulin and Mr. Alfred Salmon, was appointed to investigate the whole matter, and the system now inaugurated, which should make for the highest state of business efficiency, is the result of their deliberation. Mr. W. T. Bates has been appointed chief cashier.

